# Establishment of an Olfactory Region-specific Intranasal Delivery Technique in Mice to Target the Central Nervous System

**DOI:** 10.3389/fphar.2021.789780

**Published:** 2022-01-10

**Authors:** Johannes Flamm, Sunniva Hartung, Stella Gänger, Frank Maigler, Claudia Pitzer, Katharina Schindowski

**Affiliations:** ^1^ Institute of Applied Biotechnology, Biberach University of Applied Sciences, Biberach, Germany; ^2^ Faculty of Natural Science, University of Ulm, Ulm, Germany; ^3^ Medical Faculty, University of Ulm, Ulm, Germany; ^4^ Interdisciplinary Neurobehavioral Core, Heidelberg University, Heidelberg, Germany

**Keywords:** nose to brain drug delivery, intranasal delivery, delivery method optimisation, animal model, 3 R rule, C57BL/6

## Abstract

We have recently developed a region-specific catheter-based intranasal application method in mice by using CT scan-based 3D cast models of the murine nose (DOI: 10.2376/0005-9366-17,102). This technique is able to specifically deliver drugs to the olfactory region or to the respiratory region only. Thereby, intranasally administered drugs could be delivered either via neuronal connections to the central nervous system or via the well-perfused rostral parts of the nasal mucosa to the systemic circulation. In the present study, we transferred successfully this novel delivery technique to C57Bl/6 mice and determined parameters such as insertions depth of the catheter and maximum delivery volume in dependence to the weight of the mouse. Breathing was simulated to verify that the volume remains at the targeted area. A step-by-step procedure including a video is presented to adopt this technique for standardized and reproducible intranasal central nervous system (CNS) delivery studies (DOI : 10.3390/ pharmaceutics13111904).

## Introduction

Intranasal drug delivery has gained increasing interest as a route for drug administration, in particular for targeting of the central nervous system (CNS). However, the nasal cavity is not a uniform structure as it is covered by four different types of epithelia, the respiratory epithelium and the olfactory epithelium, but also squamous and transitional epithelium (Doty, 2003). The respiratory mucosa consists of respiratory epithelium and *lamina propria* with glands and immune cells, is well-perfused and covers about 90% of the lower nasal cavity in humans and about 50% of the rostral parts in rodents (Lochhead and Thorne, 2012). Consequently, the respiratory mucosa is an easily accessible region for nasal sprays. In fact, it has already been used in clinical practice for the targeting of drugs into the systemic circulation (Illum, 2012; Stützle et al., 2015; Gänger and Schindowski, 2018). In contrast, the olfactory mucosa that contains the olfactory epithelium is a rather well-hidden structure that is harder to access in both humans and rodents. In humans, it is located at the roof of the nasal cavity where it makes up less than 10% of the total mucosa surface (Carmichael et al., 1994). In rodents, however, it covers up to 40% of the caudal side of the rodent nasal cavity (Carmichael et al., 1994). The olfactory epithelium is composed of olfactory sensory neurons, sustentacular or supporting cells and basal cells. The axons of the olfactory sensory neurons form neuronal bundles that extend from the *lamina propria* to the olfactory bulb and provide a direct connection between the olfactory mucosa and the CNS (Gänger and Schindowski, 2018). It has previously been reported that small molecule drugs and biopharmaceuticals delivered to the olfactory mucosa can pass the so-called nose-brain barrier and reach the olfactory bulb, from where they can diffuse to other parts of the brain (Kumar et al., 2018; Maigler et al., 2021). Thus, it is believed that the olfactory mucosa mediates predominantly drug delivery to the CNS while the respiratory mucosa mediates drug absorption into the blood stream. Hence, nose-to-brain (N2B) delivery via the olfactory mucosa may be a suitable delivery route for bypassing the blood brain barrier (Illum, 2004; Lochhead and Thorne, 2014; Engelhardt et al., 2016).

**FIGURE 1:**
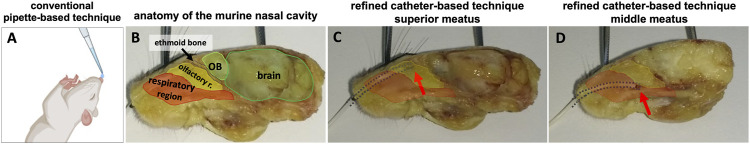
Refined catheter-based intranasal technique. **(A)** The sketch displays the conventional pipette-based technique to administer drops onto, or into, the nostril. This method is unable to discriminate the nasal regions and additionally holds a high risk for swallowing and ingestion of the drug solution. **(B)** Anatomy of a sagittal section though a murine head showing the nasal cavity with the respiratory and the olfactory regions. The ethmoid bone separates the nasal cavity from the brain (for a better visibility the structure was encircled with a dotted line). Located caudally from the ethmoid bone are the olfactory bulbs (OB) that receive neuronal connections from the olfactory mucosa through the ethmoid bone. **(C)** The recently developed refined technique uses a catheter for neonates to enter the nasal cavity via the nostrils. The correct angle for insertion through the nostrils is important to reach to dorsal meatus and, hence, to reach the olfactory region with the ethmoid turbinates (arrowhead). **(D)** A too low angle during insertion results in targeting the middle meatus (arrowhead). It is impossible to reach the olfactory region from here. Therefore, a sufficient training of the technique is required to target the olfactory region in alive animals. For a better visibility the catheter tip was encircled with a dotted line and the olfactory and respiratory regions were colored in **(C)** and **(D)**. Sketch **(A)** was created under license with Biorender.

In order to use the great potential of intranasal N2B drug delivery, many aspects are yet to be elucidated. For a successful clinical translation, quantitative studies on efficacy, safety, reproducibility, pharmacokinetics, and drug transport mechanisms are needed (Stützle et al., 2015). Although there has been a rise in publications on intranasal drug delivery experiments performed in rodents, only a minority of 3.1% delivers quantitative, robust data that are suitable for a clinical development of this drug delivery route (Kozlovskaya et al., 2014). Furthermore, even though these studies may provide information on a quantitative readout, the highly heterogenous and non-standardized intranasal application procedures that are used to administer the drugs limit the interpretation of the data. For instance, one common intranasal administration approach is performed by pouring a drop of drug solution onto, or into, the nostril of a mouse or rat (Figure 1A) (Thorne et al., 1995; Francis et al., 2008; Thorne et al., 2004). In this scenario, even the administration of a 20 µL volume (the approximate volume of a common drop) leads to a flooding of the murine nasal cavity, so that the total amount of deposited drug remains unknown. Additionally, the described administration approach is accompanied by safety issues for the laboratory animals such as the swallowing and the inhalation of the administered drug solution. Notably, rodents are obligate nose breathers, but there is no study data available on the percentage of animals that were asphyxiated by the intranasal delivery of overly high volumes (Harkema et al., 2006). Finally, with the pipette-based conventional method as demonstrated in Figure 1A it is impossible to discriminate among the regions of the nose, and hence to target a specific kind of nasal mucosa (Hanson et al., 2013). As described above, the nasal region and thereby the targeted mucosa is responsible for whether the drug is directed to the systemic circulation via uptake by blood vessels, or to the CNS along neuronal connections. Consequently, the lack of a precise and region-specific application technique for rodents is a major drawback for a meaningful clinical development of N2B delivery.

For the reasons discussed above, we have previously presented a refined intranasal administration technique that uses a catheter to specifically reach the olfactory or the respiratory region (Flamm et al., 2018). Region-specific in this context means that the liquid drug solution is applied exclusively to the olfactory mucosa without wetting of the respiratory mucosa. This approach was developed and validated with 3D-printed casts of a murine nasal cavity under 3R (replace, reduce, refine) criteria.

The aim of the present study was to transfer the region-specific technique from *in vitro* (3D casts (Flamm et al., 2018)) to mice and to determine the maximum application volume as well as the suitable insertion depth of the catheter. Both criteria were investigated in dependence of the bodyweight of the individual mice. In summary, we could successfully transfer and validate the region-specific catheter-based application technique from murine 3D casts to mice and were able to determine the average volume and insertion depth for olfactory targeting. While demonstrating here the establishment of this region-specific method in mice, we could recently provide first successful proof-of-concept studies using this technique (Maigler et al., 2021).

## Materials and Methods

### Animals

10 mice have been provided by the Interdisciplinary Neurobehavioral Core (INBC), University of Heidelberg. C57Bl/6 mice at the age of 3–5 months were used for the study. The mice were housed in groups of four per cage and with food and water *ad libitum* under a standard 12 h light/dark cycle (7:00 p.m.–7:00 a.m.) with a regulated ambient temperature of 22°C and at a relative humidity of 40–50%. All procedures were conducted in strict compliance with national and international guidelines for the care and use of laboratory animals. Animal experiments were approved by the local governing body (Regierungspräsidium Karlsruhe, Germany G-92/19) and were carried out in compliance with the ARRIVE guidelines. 10 C57Bl/6 mice (6 males and four females) were sacrificed and experiments were performed with the cadavers with a *post mortem* delay below 10 min. At the time of sacrifice the mice were 3–5 months old and had a weight between 28 and 34 g.

### Determining the Refined Procedure of the Olfactory Region-Specific Intranasal Delivery in Mice

For a region-specific application at the olfactory region, different types of catheters were tested for their ability to enter the nasal cavity through the murine nostril. Since the orifice is rather small, a neonatal catheter (Nutriline, Vygon GmbH und Co. KG, Aachen, Germany) was selected with an outer diameter of 0.6 mm. The tip of the catheter was laterally covered with a thin layer of ointment base (Bepanthen Augen- und Nasensalbe, Bayer Vital GmbH, Germany) as previously determined by a study with a 3D-printed cast of the murine nasal cavity (Flamm et al., 2018). Using gentle rotational movements, the catheter was inserted into the mouse’s nostrils as far as possible. Then, the inserted part of the catheter was marked directly at the nostril. The insertion depth and the weight of the respective mouse were determined.

### Determination of the Maximal Application Volume

Within 10 min *post mortem*, the heads were removed and a sagittal incision was made that bisected the nasal cavity while preserving the nasal septum. A neonatal catheter (Nutriline, Vygon GmbH und Co. KG, Aachen, Germany) connected with a Hamilton 10 µL-syringe (VWR International GmbH, Darmstadt, Germany) was applied through the intact nostril at an angle of 20°. For this purpose, the neonatal catheter was inserted into the intact nasal passage and gently pushed forward to the ethmoid turbinates (mean ± SD: 10 ± 1 mm). To determine the maximum volume that can be applied to the olfactory region, a 0.5% fluorescein solution (0.1% in PBS pH 7.4; Sigma Aldrich, Taufkirchen, Germany) was applied in 0.5 µL increments under UV light until the fluorescence was detected in the nasal passage/nasopharynx. This volume was recorded as the maximum volume.

After application of the maximum intranasal volume, a 10 µl pipette tip was inserted into the nasopharynx, by which inhalation and exhalation could then be simulated with a pipette. For this purpose, the pipette was set to a volume of 100 μl and 20 breath cycles were simulated within 10 s. The breathing simulation ended with an inhalation. The volume of fluorescein solution in the pipette tip was quantified with a fluorescence plate reader with λex 460 nm and λem 515 nm (SpectraMax M Series Multi-Mode Microplate Reader, Molecular Devices, San Jose, United States). The corresponding volume was denominated as the inhaled volume.

### Data Analysis and Statistics

Data were correlated and mean ± SD were calculated using Prism Version 8.3.0 (GraphPad, San Diego, United States). 10 C57Bl6 mice of both sex and a mean weight of 27 g were used as test subjects.

## Results

With the aid of a 3D cast of the murine nasal cavity we have recently determined the criteria for a refined region-specific intranasal delivery technique (Flamm et al., 2018). While with the conventional pipette-based method the whole nasal cavity is targeted, our refined technique is able to discriminate between the olfactory and respiratory region (Figure 1B).

### Refined Region-Specific Intranasal Application to the Ethmoid Turbinates in Mice Using a Neonatal Catheter

**FIGURE 2:**
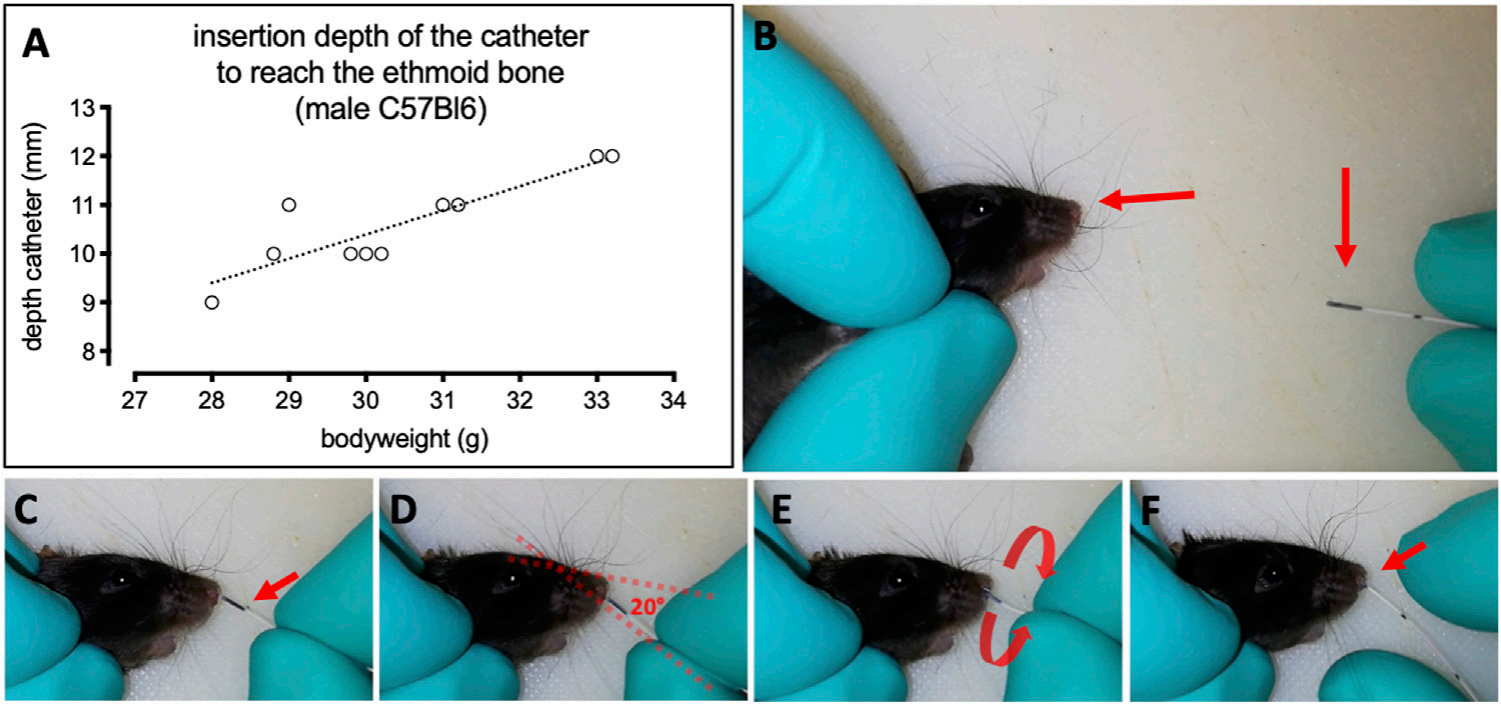
Insertion depth of the neonatal catheter and procedure of olfactory region-specific intranasal delivery in a C57Bl6 mouse cadaver. **(A)** Individual insertion depths of a neonatal catheter in the murine nose for the intranasal application at the olfactory region. The correlation between the insertion depth of the neonatal catheter and the weight of the individual animals was significant (***p < 0.0009, *r* ^2^ = 0.77, n = 10). **(B–F)** Sequence of the refined region-specific intranasal delivery targeting the olfactory region. **(B)** The mouse is kept in a supine position while the head is gently fixed with one hand. The nostril must be accessible (arrowhead) and the prepared catheter (arrowhead) kept in the other hand. **(C)** Note the colored pattern at the tip of the catheter as indicator of how far the catheter is inserted. **(D)** The catheter is carefully inserted into the nostril with a minimal angle of 20° to target the correct meatus. **(E**) During insertion the catheter must be rotated gently to advance it through the tight nasal cavity. **(F)** Once the catheter reaches the ethmoid turbinates (olfactory region) it cannot be advanced gently anymore. The colored part of the catheter is now completely introduced into the nasal cavity (arrowhead).

To transfer the methodology from the cast to real mice, 10 C57Bl6 of both sex and a mean weight of 27 g were used. The catheter application was performed post mortem. To do so, each animal’s body was placed in a supine position with the head supported at a 45- degree angle to the body (Figure 2B), and the neonatal catheter was introduced in a minimum angle of 20° and advanced with gentle rotational movements (Figures 2C–E). The application at the correct angle is important for achieving the specific targeting of dorsal meatus (Figure 1C). When a smaller angle relative to the head’s frontal line is used for introduction of the catheter, the insertion is considerably easier. However, the catheter will hereby be directed to the middle meatus of the nasal cavity (Figure 1D) and result in a misapplication.

A nasal balm applied as ointment to the sides of the catheter facilitates the insertion into the nostril and shall enable an irritation-free application in alive animals. Further, we could show in the cast models that the use of a lipophilic ointment reduced traction between the inserted catheter and the surface of the models or the mucosa, respectively. Also, the lipophilic ointment formed a barrier as it spread from the catheter tubes to the mucosa of the lower parts of the nasal cavity, and hence hampered intranasally administered hydrophilic formulations from spreading into these anterior parts of the nasal cavity.

To give optical control over the nasal cavity, sagittal sections of the heads were prepared by sagittal incision and removal of the upper tissue lateral from the nasal septum. A catheter was introduced into each individual nasal cavity as described in Figure 2 and advanced as far as gently possible to the ethmoid turbinate. Thereby, the tip of the catheter was placed into the region of the nasal cavity covered with olfactory mucosa and in close proximity to the ethmoid bone and thus in direct proximity to the olfactory bulb. The correlation of the insertion depth respective to the individual weights is presented in Figure 2A with *r* ^2^ = 0.77. The mean penetration depth of the catheter was 10.6 ± 0.97 mm (mean ± SD).

As shown in Figure 1C, the dorsal meatus is comparable to a dead end. Therefore, the catheter must not be advanced against resistance in order to avoid irritations and bleeding in alive rodents. If the catheter is accidentally inserted through the middle meatus (Figure 1D), a misapplication can be avoided by monitoring of the maximum insertion depth. In the case of a misinsertion *via* the middle meatus, the catheter can be inserted far deeper than 10 mm, as it will be pushed through the nasal passage into the nasopharynx. By monitoring the insertions depth, misinsertion of the catheter can be noticed and misapplication can be prevented by removing the catheter and reintroducing it in a correct angle.

A video demonstrating a successful application is provided as supplementary data.

### Determination of the Application Volume for a Region-Specific Delivery at the Olfactory Region

**FIGURE 3:**
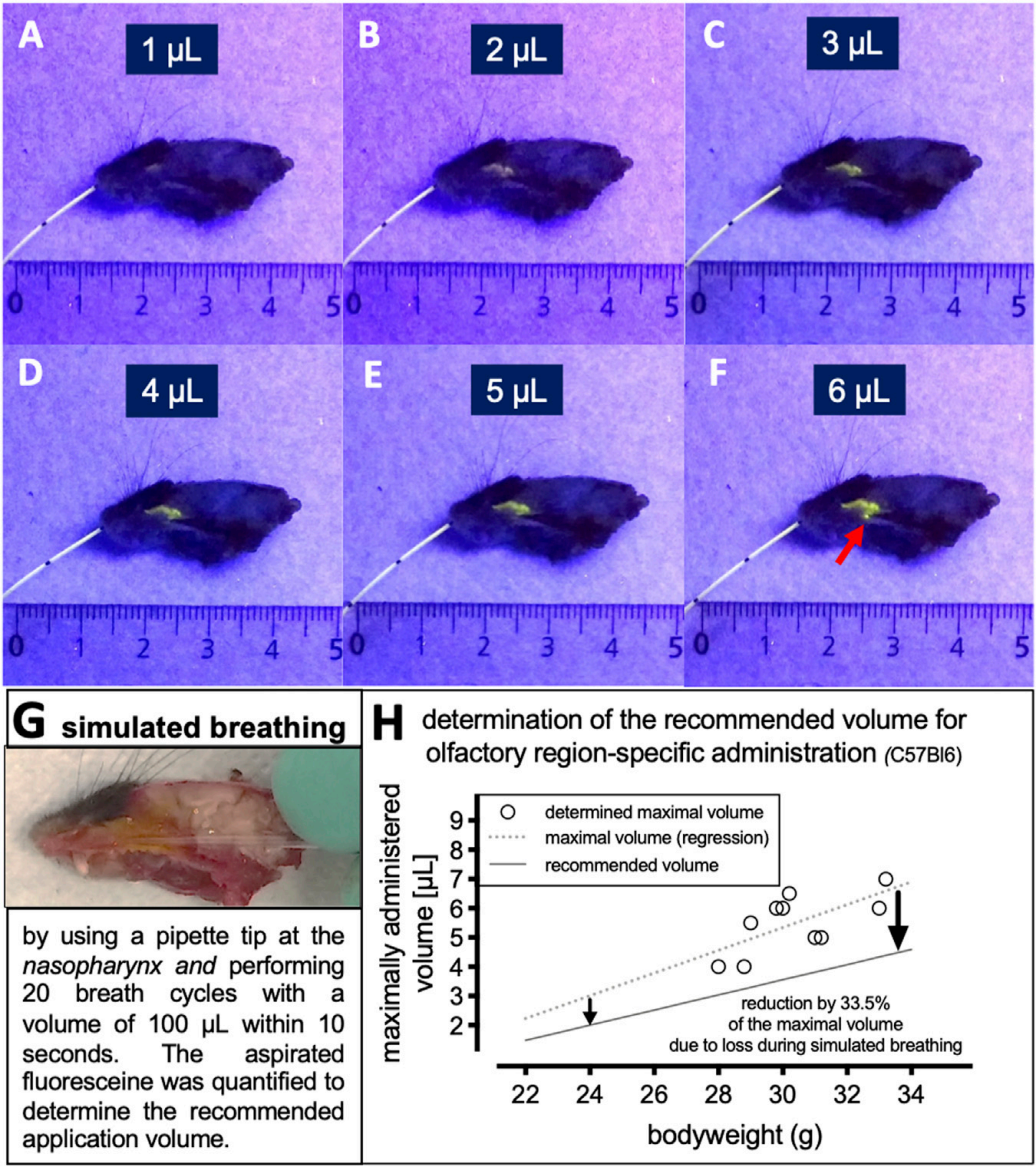
Determination of maximum volume to target the olfactory mucosa in C57Bl6 mice. **(A–E)** The parts lateral from the nasal septum had been removed carefully to give access to the olfactory region. The catheter was inserted to target the olfactory region and a fluorescein solution was applied in steps of 0.5 µL under UV illumination. **(F)** The maximum volume was determined when the fluorescein solution drained into the nasal passage as indicated with the arrowhead. This occurred during the increase of applied volume from 5.5 to 6 µL. Thus, the maximum volume for olfactory region-specific targeting was determined as 5 µL. **(G)** Insertion of a 10 µl pipette tip into the nasopharyngeal duct of the murine skull to simulate the murine breathing pattern with inhalation and exhalation. 20 simulated breath cycles with a volume of 100 µL each were performed within 10 s. The volume of fluorescein sodium that remained in the pipette after the last inhalation was quantified and denominated as the aspirated volume. **(H)** Determination of the dependency of the experimentally determined maximum volume and the recommended volume with the weight of the individual mouse cadavers. The was a significant correlation between experimentally determined volumes and individual weight (*p < 0.04, *r* ^2^ = 0.43, *n* = 10).

To guarantee administration to the olfactory region only, the applied volume must be limited to avoid drainage to other nasal areas, as well as to avoid swallowing or inhalation of the drug solution. The maximum application volume was investigated by the stepwise administration of a fluorescein solution to the olfactory region with a Hamilton syringe through the catheter. Under UV illumination, the fluorescent area was visually inspected (Figures 3A–E) and drainage to the nasal passage was considered as excessive volume (Figure 3F). A linear regression of the determined maximum volume and the individual weights of the animals is displayed with the dotted line in Figure 3H. A weak correlation with increasing weight was observed (*r* ^2^ = 0.66; n = 10).

Since isolated mouse heads were used in this study, the administered solution was not exposed to air flow or pressure originating from respiration. For a successful transfer of the technique for future *in vivo* studies, the effects of breathing must not be neglected. The mouse with an average weight of 31 g has a lung volume of approximately 150–250 µL and takes 170 to 320 breaths per minute (Moshkin et al., 2014). Therefore, we decided to approximate 100 µL as volume that flows through one nasal hemisphere and 120 breaths per minutes since for the intranasal delivery the animals will undergo anesthesia, which reduced the breathing frequency. Thus, to mimic the breathing of a mouse, a fine pipette tip was inserted into the nasopharyngeal duct of the murine skull (Figure 3G) and 20 “breaths” were simulated by pipetting a volume of 100 µL up and down within 10 s. Thereby, the air stream drives the fluid slightly back and forth and some liquid is aspirated by the pipette. The volume of the ‘inhaled’ fluorescein solution in the pipette was quantified after the last “inhalation.” The mean aspirated volume was 33.5% of the maximum volume applied and no obvious correlation with the bodyweight was identified (data not shown). To calculate the recommended volume for olfactory targeting, each individual maximum volume was reduced by 33.5% as demonstrated by the black line in Figure 3H. The recommended application volumes as presented in Figure 3H and Table 1 should not be exceeded in order to prevent distribution in the nasal passage, to the nasopharynx, but also to avoid inhalation or ingestion. It should be noted that fluoresceine solution was predominantly aspirated during the initial simulated breathing cycles and remained stable after five cycles. Therefore, the simulation was terminated after 20 cycles.

**TABLE 1:** Recommended volumes for olfactory-specific delivery in C57Bl6 mice.

Weight [g]	18	19	20	21	22	23	24	25	26	27	28	29	30	31	32	33	34
volume [µL]	0.4	0.7	1.0	1.2	1.5	1.7	2.0	2.3	2.5	2.8	3.0	3.3	3.6	3.8	4.1	4.3	4.6

## Discussion

A comprehensive refinement of the intranasal delivery in rodents has recently been demonstrated based on 3D anatomical cast models of the murine nose and nasal cavity (Flamm et al., 2018). The use of a catheter instead of a pipette tip for delivery enables region-specific targeting (Hanson et al., 2013; Gänger and Schindowski, 2018). Thereby, the exclusive targeting of the olfactory region for a standardized, reproducible N2B delivery becomes feasible. Also, the usage of the catheter in combination with a lipophilic ointment (petroleum jelly or a dexpanthenol-containing nasal balm) was shown to reduce the traction and to limit the spreading of the applied drug to the lower parts of the nasal cavity. Although there has been remarkable progress in the accuracy of 3D printing and prototyping processes in the last few years (Liska et al., 2007), the scanning and printing of small structures such as murine nasal turbinates and meatuses still proves to be a challenge. A further issue with considerable influence on the characteristics of a 3D printed prototype is the choice of material used in the printing process (Flamm et al., 2018). While a transparent and flexible silicone cast successfully mimics the cartilage-like texture of the murine skull, the hydrophobic surface characteristics of silicone are far from that of a mucosal surface. Nevertheless, the use of 3D printing is a promising approach to refine and advance drug delivery technologies under the 3R criteria (Engelhardt et al., 2016). Despite the above-mentioned limitations, the murine 3D casts have provided a good basis for the development and training of the catheter-based refined technique. In the present study, we have transferred this refined application method to mice and determined the relevant parameters for a standardized and reproducible methodology for future pharmacokinetic and bioactivity studies.

During the intranasal application, the nostril of the mouse poses the first important hurdle as it is the narrowest structure to be passed. Here, we suggest to use an angle of 20-25° relative to the noseline and to push the catheter forward by gentle rotations (Figures 2C–E). The mean penetration depth of the catheter was marginally longer in the application in the mouse than what was determined previously in the 3D printed casts. The differences in insertion depth may be due to the material properties of the 3D-printed casts. Probably, the delicate and fine structures of the ethmoid turbinates as they occur in mice could not be accurately resolved by the CT scans of the mice, or during the prototyping of the casts. Importantly, we suggest to use the insertion depth of the catheter respective to the mice’s weight as a means to control the application (Figures 1C,D and Figure 2A). While the penetration depth is limited when the catheter is inserted to the dorsal meatus (Figure 2F), an insertion to the middle meatus directs the catheter through the nasal passage further down to the lower airways or the esophagus, so that no apparent limitation in insertion depth occurs. By controlling the insertion depth, misapplications can be avoided.

As demonstrated by the sagittal sections prepared from the murine heads (Figures 3A–F), the refined technique resulted in a region-specific application to the ethmoid turbinate, which is the major part of the olfactory region (Doty, 2003). The rostral parts of the murine nasal cavity, which are predominantly covered with respiratory mucosa, did not display any fluorescence when the fluorescein solution was administered to the ethmoid turbinates (Figures 3A–F). The use of a lipophilic ointment achieved that not even traces of the fluorescein solution could appear at the respiratory regions, despite this area being part of the catheter’s path. Dexpanthenol-containing nasal balms are already approved for clinical use at the nasal mucosa and are hence as an ointment well suitable for the refined technique in rodents. The balm used in this study contains paraffin and petroleum jelly, which are both of hydrophobic character. Consequently, the ointment is able to hinder hydrophilic formulations and water-based solutions from spreading and prevents them from draining to the lower parts of the nasal cavity covered with respiratory mucosa (Peltier et al., 2020). Other formulations such as water-based hydrogels would reduce the surface tension of the water-based drops at the tip of the catheter and, hence, could possibly drain to the rostral parts covered with respiratory mucosa. In a recent study we demonstrated that a region-specific administration is highly important for CNS targeting (Maigler et al., 2021). Finally, the ointment also serves as lubricant to avoid irritations and lesions on the sensitive mucosa that may be caused by the catheter.

The hydrophobicity of the 3D casts’ surfaces was the reason for why it has not been feasible to determine the influence of breathing on the distribution of the intranasally applied solutions with the 3D printed prototypes. Preliminary unpublished data showed that the surface tensions of the administered drops rendered the spreading and wetting of the applied drug solution on the silicone surface as almost impossible. Therefore, any airstream applied with the aim of simulating the breathing pattern of a mouse in the 3D casts resulted in either a “sneezing”- or an “inhaling”-like behavior of the administered drops. Hence, the effect of breathing activity on intranasal drug distribution could not adequately be investigated in the 3D cast. Instead, the influence of breathing was now simulated in prepared mouse heads (Figure 3G) to determine the maximum delivery volume at the olfactory region and to avoid overapplication. Previous computer-based airflow simulations studies suggested that airflow in the murine olfactory region is mostly unidirectional (Coppola et al., 2017; Coppola et al., 2019). During inspiration, the air flows into the olfactory region *via* the dorsal meatus and then exits ventrally at the location of the nasopharynx. During expiration, the flow in the olfactory region is mostly stagnant, indicating that the olfactory region is continuously purged in a unidirectional manner. Although, in the present study the simulated aspiration of the fluoresceine solution occurred mainly during the first three initial simulated breathing cycles, we cannot exclude that the here used breathing simulation did not perfectly mimic the purged volume under *in vivo* conditions, which depends on the inspiratory airflow rate and the number of respiratory cycles. Probably, the remaining fluoresceine solution was trapped in the olfactory region due to high surface tension forces. Last but not least, in a recent *in vivo* study in mice we did not observe any significant levels of ingested fluoresceine with the here described technique while ingested fluoresceine was found with the pipette-based method (Maigler et al., 2021).

As expected, the maximum applicable volume exceeds the volume that remains in the olfactory region during breathing. The experimentally derived recommended volumes for intranasal application with the refined technique are displayed in relation to the mouse’s weight in Figure 3H and in Table 1. It should be noted that a suitable syringe is needed to apply such small volumes accurately. The parameters used in the present study correspond to the physiological conditions that occur in mice with an average weight of 31 g (Moshkin et al., 2014). It should be noted that the breathing frequency and intensity may change with stress or anesthesia during animal experimentation. Moshkin and co-workers determined the intranasal volume from one hemisphere of a mouse nose using magnetic resonance imaging and obtained a volume of 17 µL. The higher volume can be accounted to the fact that the anterior nasal cavity (covered with respiratory mucosa) was not included in our experiments. However, it can be assumed that volumes of at least 20 µL are delivered by the pipette-based method (Figure 1A), which will undoubtedly target the respiratory as well as the olfactory mucosa and will be swallowed, inhaled or exhaled, which altogether makes it impossible to generate reliable quantitative data (Coppola et al., 2014; Kozlovskaya et al., 2014). The here presented region-specific intranasal technique allows the administration of a small volume to the ethmoid turbinates, thanks to which it prevents the discussed issues of the conventional method and enables the performance of standardized and reproducible intranasal N2B studies. Finally, the here described technique has proven itself as successful in intranasal *in vivo* studies (Maigler et al., 2021).

As recently demonstrated, N2B transport via the olfactory region is an application route that allows hydrophilic and large molecules to enter the CNS by bypassing the blood-brain barrier (Maigler et al., 2021). In contrast, small lipophilic molecules (<500 Da) often cross the blood-brain barrier by diffusion. Therefore, our refined intranasal delivery technique via the olfactory region is highly suitable for larger hydrophilic drugs such as proteins. However, when administering lipophilic drugs to the olfactory mucosa, the drug formulation should be adapted to the pharmacokinetic properties of the drug. An oil-in-water (o/w), a water-in-oil (w/o) emulsion or a flowable cream with these properties could be suitable for lipophilic drugs. Presumably, it would be useful to substitute the here described hydrophobic dexpanthenol-containing ointment with a hydrophilic liquid or semi-solid formulation such as a Carbomer-based hydrogel. In this case, drug absorption via the respiratory region would also be reduced. However, the mucus layer covering the respiratory mucosa already has a mucin-based hydrogel so the effect should be less pronounced.

Last but not least, the here described delivery method also enables the region-specific delivery of viscous formulations, such as hydrogels. While the pipette-based method can only apply formulations with a low viscosity to the olfactory region by filling up the entire nasal cavity, our novel technique makes it possible to exclusively reach the ethmoid turbinates with gel formulations. Nevertheless, the application of gels must be performed carefully, slowly and steadily. This allows the gel emerging from the application device to gradually distribute in the nasal cavity, which ensures that little pressure is exerted on the nasal septum. Finally, when applying gels, it should be considered that mice are obligate nasal breathers (Harkema et al., 2006). In this context, care must be taken that the applied gel does not occlude the nasal airways. Therefore, it seems necessary to limit the application of intranasal gel formulations to one nasal hemisphere.

## Data Availability

The original contributions presented in the study are included in the article/Supplementary Material, further inquiries can be directed to the corresponding author.
